# Deriving a mapping algorithm for converting SF-36 scores to EQ-5D utility score in a Korean population

**DOI:** 10.1186/s12955-014-0145-9

**Published:** 2014-09-24

**Authors:** Seon-Ha Kim, Seon-Ok Kim, Sang-il Lee, Min-Woo Jo

**Affiliations:** Department of Nursing, Dankook University, 119 Dandaero, Cheonan, 330-714 South Korea; Department of Clinical Epidemiology and Biostatistics, Asan Medical Center, 86, Asanbyeongwon-gil, Songpa-gu, 138-736 Seoul South Korea; Department of Preventive Medicine, University of Ulsan College of Medicine, 86, Asanbyeongwon-gil, Songpa-gu, 138-736 Seoul South Korea

**Keywords:** EQ-5D, SF-36, Quality of life, Utility, Korea

## Abstract

**Background:**

There is no research on mapping algorithms between EQ-5D and SF-36 in Korea. The aim of this study was to derive a predictive model for converting the SF-36 health profile to the EQ-5D index using data from several studies.

**Methods:**

Individual data (n = 2211) were collected from three different studies and separated into derivation (n = 1660) and internal validation sets (n = 551). Data from 123 colon cancer patients were analyzed for external validation. The prediction models were analyzed using ordinary least-square (OLS) regression, two-part modeling, and multinomial logistic modeling using eight scale scores; two summary scores and the interaction terms of SF-36 were used as independent variables. The EQ-5D index using the Korean value set and each dimension of the EQ-5D were used as dependent variables. The mean absolute errors (MAE) and R^2^ values of the internal and external validation dataset were used to evaluate model performance.

**Results:**

Our findings show that the three different scoring algorithms demonstrate similar performances in terms of MAE and R^2^. After considering familiarity and parsimony, the OLS model (including Physical Function, Bodily Pain, Social Function, Role Emotional, and Mental Health) was found to be optimal as the final algorithm for use in this study. The MAEs of the OLS models demonstrated consistent results in both the derivation (0.087–0.109) and external validation sets (0.082–0.097).

**Conclusion:**

This study provides mapping algorithms for estimating the EQ-5D index from the SF-36 profile using individual data and confirms that these algorithms demonstrate high explanatory power and low prediction errors.

## Introduction

Quality-adjusted-life year (QALY) is a single measure that combines reduced morbidity (quality gains) and reduced mortality (quantity gains) [1]. Cost-utility analysis in economic appraisal was developed to compare the costs of a healthcare program and its beneficial impacts on both length and quality of life [2]. Calculating QALYs requires quality weights for each health state. Several multi-attribute utility instruments and quality weight tariffs are available: EuroQol-5 dimension (EQ-5D) [3], Health Utilities Index Mark 2 and 3 [4,5], Quality of Well-being Scale [6], and short form (SF)-6D [7]. Many countries have derived country-specific utility weights, and there is some evidence that the value sets between countries are substantially different [8]. Therefore, mapping algorithms developed in other countries might be inappropriate for Korean-specific decision making. Converting algorithms from generic Health-related Quality of Life (HRQOL) measures to preference-based measures is an increasingly common solution when health utility values are unavailable for cost-utility analysis. EQ-5D utility weights are already relatively common in South Korea [9,10]. SF-36 is one of the most popular generic instruments for measuring HRQOL, and SF-36 descriptive data are often available. Psychometric properties of SF-36 Korean version in general population has been demonstrated [11,12]. SF-6D was developed as a preference-based measure that uses either SF-36 or SF-12 [7,13]; however, no algorithm exists in Korea for converting SF-36 to SF-6D. Therefore, Korean-specific mapping algorithms for converting SF-36 to EQ-5D utility index are needed.

Several algorithms for converting the SF family of instruments to EQ-5D have been introduced [14-16], including ordinary least-square (OLS) regression, multinomial logistic (MNL) regression, and censored least absolute deviation (CLAD) regression. The independent variables, two summary scores, eight domain scores, and item responses included on the SF family instrument are used in these algorithms. There is no standard mapping technique that can translate SF-36 to the EQ-5D utility index. OLS regression is one of most frequently used mapping approaches because of its applicability and interpretability. The two-part approach consists of logistic and least-square regressions that model specific features of the EQ-5D index, such as ceiling effect and other data included in the EQ-5D index [17]. A variety of mapping method to convert SF data to EQ-5D demonstrated inconsistent results in previous studies. Chuang & Kind suggested that OLS regression is more accurately estimates group mean than MNL, CLAD, and two-part modeling [14]. Rowen et al. reported that random-effects Generalized Least Squares demonstrates more accurate predictions than Tobit or CLAD [18]. On the other hand, Sullivan & Ghushchyan reported that the CLAD demonstrates the lowest mean predictive error, followed by OLS and Tobit [19]. Le & Doctor reported that Bayesian networks consistently outperform other mapping models, including MNL, OLS, and CLAD [20].

This study explores mapping algorithms for converting SF-36 to the Korean EQ-5D index using three different techniques: OLS regression, MNL regression, and two-part modeling.

## Methods

### Datasets

Individual-level data (n = 2211) were collected from three published studies and randomly divided into derivation (n = 1660) and internal validation sets (n = 551). These three studies included patients from the general population [21], type 2 diabetic patients visiting outpatient clinics at three university hospitals [22], and stroke patients in a single community [23]. Survey data that measured HRQOL in colon cancer patients (n = 123) was also used for external validation [24]. Study on general population, type 2 diabetic patients, stroke patients and colon cancer patients were conducted in 2011, 2007, 2008 and 2010, respectively. Further details are elsewhere [21-24].

### Instruments

All surveys included both the EQ-5D and SF-36 questionnaires. EQ-5D is a generic preference-based measure that describes health status according to five dimensions: mobility, self-care, usual activities, pain discomfort, and anxiety/depression. Each dimension is scored accordingly: no problem, some or moderate problems, or extreme problems [25]. The EQ-5D utility index was calculated using the valuation set from the Korean population [10]. Therefore, possible EQ-5D scores range from −0.171 to 1.0, with 1.0 denoting “full health” (11111 state) and 0.0 denoting “death”.

SF-36 is a generic health measure that consists of 36 items with 3–6 levels. The SF-36 health profiles measure eight health domain scores (physical functioning [PF], role-physical [RP], role-emotional [RE], bodily pain [BP], general health [GH], vitality [VT], mental health [MH], and social functioning [SF]) and two summary scores (physical component summary [PCS] and mental component summary [MCS]) [26]. Each raw domain scores can be converted to a 0–100 scale, where a higher score indicates a higher health status.

### Analysis

Three approaches—OLS regression, two-part modeling, and MNL modeling—were used to develop a mapping algorithm for converting SF-36 to EQ-5D.

### OLS regression

OLS chooses regression coefficients in order to minimize the sum of the squares of the errors. A recent mapping review reported that the most common mapping method was OLS [27]. However, The OLS model does not restrict the range of values and therefore may lead to implausible predicted values outside of the existing range of the EQ-5D values [28]. We used OLS regression with the sandwich variance estimator in order to account for the clustering effects of communities and hospitals.

### Two-part modeling

Two-part modeling is recommended because of the specific features of the EQ-5D index described above [29]. This model divides the study population accordingly: people who report a full health state on EQ-5D (i.e., 11111), and people who had > 1 problem on any of the five dimensions on EQ-5D. The first part of the model consists of logistic regression, which is used to determine the probability of achieving the maximum EQ-5D index score of 1.0. The second part is least-square regression with robust variance estimation of the EQ-5D scores, which is performed on the subset of patients whose EQ-5D score is not equal to 1.0 [17].

### MNL modeling

The MNL model estimates a particular level for each EQ-5D dimension rather than using the EQ-5D index score. We used the MNL model for each EQ-5D dimension to derive the probability that the dimension was at level 1, 2 or 3, and then Monte Carlo simulation was used to generate random number (u_i_) between 0 and 1 [30]. We performed multiple Monte Carlo simulations using derivation set, but the results are similar, and so we generated random variables by a single simulation. Here, P_1_(X_j_), P_2_(X_j_), and P_3_(X_j_) indicate the predicted probabilities of MNL regression for response levels 1, 2, and 3, respectively, where X_j_ represents the each EQ-5D domain. A response level for each of the EQ-5D domains was assigned as follows using P_1_(X_j_), P_3_(X_j_) and u_i_ generated from simulation [20]:

$$ \mathrm{Predicted}\ \mathrm{E}\mathrm{Q}-5\mathrm{D}\ \mathrm{response}\ \mathrm{level}=\begin{array}{l}1\ \mathrm{if}\ {\mathrm{u}}_{\mathrm{i}}\le {\mathrm{P}}_1\left({\mathrm{X}}_{\mathrm{j}}\right)\hfill \\ {}2\ \mathrm{if}\ {\mathrm{P}}_1\left(\mathrm{X}\right)<{\mathrm{u}}_{\mathrm{i}}\le \left[1-{\mathrm{P}}_3\left({\mathrm{X}}_{\mathrm{j}}\right)\right]\hfill \\ {}3\ \mathrm{if}\ {\mathrm{u}}_{\mathrm{i}}>\left[1-{\mathrm{P}}_3\left({\mathrm{X}}_{\mathrm{j}}\right)\right]\hfill \end{array} $$

Using estimated responses across all five dimensions, a health state and index score can be determined according to the Korean EQ-5D value set [10].

### Model specification

We assessed the two approaches for assessing independent variables that are described in previously reported studies [14,15,18]. One model used eight raw scale scores from SF-36, and the other used two summary measures (PCS and MCS) with or without the square term and demographic variables (e.g., sex, age, education level, marital status). The dependent variable was the EQ-5D utility score. Models for use in OLS regression were selected using the backward elimination method, and *p <* 0.05 was considered statistically significant. OLS models were estimated using the following: (1) all eight scales; (2) backward elimination of all eight scales; (3) backward elimination of all eight scales and their squared terms; (4) backward elimination of all eight scales, their squared terms, and demographic factors; (5) two summary measures; and (6) two summary measures, their squared terms, and their interaction terms. The independent variables in models 1, 2, 3, and 6 were used in MNL and two-part modeling.

To compare models, we considered goodness-of-fit, applicability, and parsimony. Goodness-of-fit represents how well the model explains the observed data. We examined these models using residual diagnostic plots. Mean absolute error (MAE)—the average of the absolute differences between observed and predicted values— and root mean squared error (RMSE) were considered an important indicator during model selection. Small MAE indicates a better model. Proportions of estimation with absolute error > 0.05 and absolute error > 0.1 were also assessed. R^2^ on OLS regression, pseudo R^2^ on MNL, the mean of the estimated EQ-5D index score, and the ranges of both the derivation and validation sets were computed. Finally, practical applicability and model simplicity were considered if the models demonstrated similar MAE and R^2^ values.

All statistical analyses were conducted using SAS (ver. 9.1; SAS institute Inc., Cary, NC).

## Results

### Demographic characteristics

The total number of individuals used in the derivation, internal, and external validation sets were 1660, 551, and 123, respectively. The demographic characteristics and health status for these three sets are presented in Table 1. The average age of the derivation set was 56.9 years (SD = 15.0), 44.8% were female, and the average EQ-5D index score was 0.816 (SD = 0.266). There are no significantly different variables between the derivation and internal validation sets, whereas significant differences in the EQ-5D index, PF, GH, VT, MH, and PCS scores between the derivation and external validation sets shows that the respondents in the external validation set tended to be healthier than those in derivation set.

**Table 1:** **Demographic characteristic and health states of the included patients**

**Variables**	**Derivation set**								**Internal validation set**		**External validation set**	
	**Total**		**General population**		**Diabetes mellitus**		**Stroke**					
n	1660		448		770		442		551		123	
Age (y), mean (SD)	56.9	(15.0)	44.5	(15.5)	57.6	(11.9)	68.3	(8.2)	58.3	(14.5)	57.1	(10.0)
Female (%)	44.8		50.2		44.4		39.8		44.7		36.6	
Education (%)												
Elementary	30.9		8.5		25.1		63.6		33.2		-	
Middle school	13.5		9.2		16.4		12.9		11.3		-	
High school	33.4		47.5		33.2		19.5		34.1		-	
College or higher	22.2		34.8		25.3		4.1		21.4		-	
Marital status, married (%)	72.5		70.3		77.3		70.3		75.8		-	
EQ-5D index, mean (SD)	0.816	(0.266)	0.945	(0.091)	0.914	(0.124)	0.513	(0.325)	0.831	(0.236)	0.871	(0.113)
EQ-5D profile, 11111(%)	43.2		65.4		53.1		3.4		43.2		30.1	
EQ-5D profile, 33333(%)	1.7		0		0.1		6.1		0.9		0	
SF-domain scores, mean (SD)												
PF	66.1	(34.8)	86.6	(21.8)	77.8	(23.8)	25.0	(27.0)	65.9	(34.2)	72.1	(22.6)
RP	68.7	(35.8)	89.0	(20.4)	79.2	(27.8)	29.8	(30.6)	69.3	(34.8)	65.4	(29.3)
BP	72.3	(28.9)	84.4	(22.1)	77.6	(26.9)	50.8	(27.1)	71.5	(29.2)	76.4	(24.5)
GH	49.1	(23.9)	66.3	(19.7)	50.5	(20.5)	29.2	(18.2)	49.2	(23.7)	56.6	(20.0)
VT	40.7	(21.4)	54.1	(16.2)	42.8	(20.1)	23.3	(16.3)	39.7	(21.1)	47.4	(18.7)
SF	73.4	(32.0)	89.0	(18.5)	84.4	(22.5)	38.4	(30.6)	73.3	(31.2)	75.0	(24.6)
RE	73.8	(34.0)	89.8	(18.9)	83.8	(25.1)	40.2	(36.5)	74.8	(32.5)	71.7	(29.7)
MH	66.0	(24.7)	76.9	(17.7)	72.1	(21.4)	44.5	(44.5)	66.2	(24.6)	70.1	(19.7)
SF summary score, mean (SD)												
PCS	44.7	(11.7)	52.2	(8.0)	47.4	(9.2)	32.3	(9.1)	44.5	(11.5)	46.5	(8.0)
MCS	43.9	(13.1)	49.9	(8.3)	47.3	(10.8)	31.9	(13.0)	44.0	(12.8)	44.9	(11.0)

PF, physical functioning; RP, role- physical; BP, bodily pain; GH, general health; VT, vitality; SF, social functioning; RE, role -emotional; MH, mental health; PCS, physical component summary; MCS, mental component summary.

### OLS regression performance

The results of the OLS regression analysis are shown in Table 2. In the derivation set, R^2^ values ranged between 0.680–0.750. All OLS models predicted the average EQ-5D index, however the upper limits of estimation for all OLS models (except model 6) exceeded the upper limit of the EQ-5D index (i.e., 1). The coefficients of RP, GH, and VT in model 1 were not statistically significant. Among all models, models 3 and 4 demonstrated the lowest MAE values (0.087) and proportions of estimation with absolute error >0.05 or absolute error > 0.1 were lower than the other OLS models. Similar findings were observed in the internal and external validation sets. Demographic factors were not statistically significant (except age).

**Table 2:** **Ordinary least-square regression modeling performed using main effects with or without significant demographic and squared terms**

		**Model 1**		**Model 2**		**Model 3**		**Model 4**		**Model 5**		**Model 6**	
		**β**	**SE**	**β**	**SE**	**β**	**SE**	**β**	**SE**	**β**	**SE**	**β**	**SE**
**Derivation set**													
Intercept		**0.0161**	0.1019	**0.0168**	0.0999	−0.7629	0.1066	−0.6947	0.0861	0.9277	0.0040	0.9582	0.0059
PF		0.0179	0.0030	0.0180	0.0027	0.0696	0.0091	0.0720	0.0092				
RP		**0.0010**	0.0014										
BP		0.0051	0.0013	0.0050	0.0009	0.0114	0.0007	0.0115	0.0007				
GH		**0.0003**	0.0004			0.0029	0.0011	0.0024	0.0010				
VT		**−0.0018**	0.0021										
SF		0.0162	0.0024	0.0166	0.0022	0.0656	0.0086	0.0644	0.0082				
RE		0.0102	0.0040	0.0107	0.0036	0.0432	0.0054	0.0418	0.0055				
MH		0.0050	0.0005	0.0043	0.0013	0.0058	0.0011	0.0058	0.0011				
PF squared						−0.0013	0.0002	−0.0014	0.0002				
SF squared						−0.0042	0.0005	−0.0041	0.0005				
RE squared						−0.0018	0.0002	−0.0017	0.0002				
Age								−0.0011	0.0003				
PCS										0.1227	0.0182	0.0509	0.0046
MCS										0.0769	0.0128	0.0196	0.0029
PCS × PCS												−0.0265	0.0037
MCS × MCS												−0.0113	0.0015
PCS × MCS												−0.0334	0.0034
R^2^		0.6807		0.6804		0.7476		0.7498		0.6366		0.7093	
MAE		0.101		0.101		0.087		0.087		0.109		0.094	
AE > 0.05 (%)		53.1		52.7		49.6		50.0		62.8		50.4	
AE > 0.1 (%)		33.9		34.3		28.0		28.4		36.8		29.3	
RMSE		0.150		0.150		0.134		0.133		0.160		0.143	
EQ-5D index	Actual	Predict		Predict		Predict		Predict		Predict		Predict	
Mean (SD)	0.816 (0.266)	0.816 (0.220)		0.816 (0.220)		0.816 (0.230)		0.816 (0.230)		0.816 (0.212)		0.816 (0.224)	
Min/max	−0.171/1	0.291/1.057		0.293/1.049		0.099/1.020		0.091/1.037		0.285/1.111		−0.002/0.997	
**Internal validations set**													
MAE		0.103		0.103		0.091		0.088		0.108		0.096	
AE > 0.05 (%)		55.5		55.7		47.9		51.2		61.5		51.4	
AE > 0.1 (%)		35.4		37.0		29.8		29.4		36.5		30.0	
RMSE		0.147		0.147		0.132		0.131		0.155		0.144	
EQ-5D index	Actual	Predict		Predict		Predict		Predict		Predict		Predict	
Mean (SD)	0.831 (0.236)	0.809 (0.217)		0.809 (0.217)		0.824 (0.217)		0.812 (0.228)		0.819 (0.201)		0.822 (0.208)	
Min/max	−0.171/1	0.294/1.059		0.293/1.049		0.143/1.015		0.113/1.040		0.343/1.104		0.131/0.993	
**External validation set**													
MAE		0.092		0.082		0.085		0.086		0.097		0.083	
AE > 0.05 (%)		61.0		61.0		56.1		61.0		66.7		56.1	
AE > 0.1 (%)		35.8		35.0		30.9		33.3		41.5		26.8	
RMSE		0.123		0.123		0.114		0.115		0.126		0.115	
EQ-5D index	Actual	Predict		Predict		Predict		Predict		Predict		Predict	
Mean (SD)	0.871 (0.113)	0.840 (0.149)		0.842 (0.150)		0.894 (0.125)		0.893 (0.121)		0.846 (0.156)		0.875 (0.129)	
Min/max	0.537/1	0.432/1.045		0.430/1.049		0.418/1.032		0.416/1.023		0.435/1.111		0.355/0.984	

PF, physical functioning; RP, role- physical; BP, bodily pain; GH, general health; VT, vitality; SF, social functioning; RE, role -emotional; MH, mental health; PCS, physical component summary; MCS, mental component summary; MAE, mean absolute error; AE, absolute error; RMSE, root mean squared error.

Bold values are not significant (*p* > 0.05).

### Two-part modeling performance

The performance of the two-part model is described Table 3. Models 7, 8, 9, and 10 used the same independent variables as models 1, 2, 3, and 6, respectively. The predicted mean EQ-5D indexes of models 7, 8, 9 and 10 were 0.829, 0.829, 0.829, and 0.828 respectively, which are slightly higher than the actual EQ-5D index of 0.817. According to the two-part model, the upper and lower boundaries of the predicted EQ-5D are lower than the OLS models. Of the included two-part models, model 9 demonstrated the lowest MAE value of 0.081 in the derivation set; on the other hand, the external validation set demonstrated the MAE value of 0.086.

**Table 3:** **Two-part modeling (logistic + ordinary least-square regression): predicting EQ-5D index scores from SF-36**

	**Model 7** ^**a**^		**Model 8** ^**b**^		**Model 9** ^**c**^		**Model 10** ^**d**^	
**Derivation set**								
MAE	0.089		0.089		0.081		0.085	
AE > 0.05 (%)	50.3		49.7		47.6		48.6	
AE > 0.1 (%)	31.9		31.9		29.3		32.2	
RMSE	0.298		0.299		0.284		0.292	
Predicted EQ-5D index								
Mean (SD)	0.829	(0.233)	0.829	(0.233)	0.829	(0.239)	0.828	(0.234)
Min/max	0.193	1.078	0.198	1.079	0.076	1.000	−0.088	1.000
**Internal validation set**								
MAE	0.090		0.089		0.083		0.090	
AE > 0.05 (%)	50.8		52.3		49.7		50.8	
AE > 0.1 (%)	34.3		47.7		30.3		35.2	
RMSE	0.301		0.303		0.289		0.300	
Predicted EQ-5D index								
Mean (SD)	0.823	(0.231)	0.829	(0.233)	0.825	(0.237)	0.826	(0.229)
Min/max	0.222	1.082	0.198	1.079	0.102	1.000	−0.057	1.000
**External validation set**								
MAE	0.091		0.091		0.086		0.081	
AE > 0.05 (%)	57.7		59.4		59.4		53.7	
AE > 0.1 (%)	38.2		39.0		38.2		34.2	
RMSE	0.302		0.302		0.293		0.285	
Predicted EQ-5D index								
Mean (SD)	0.885	(0.113)	0.881	(0.154)	0.908	(0.125)	0.890	(0.129)
Min/max	0.377	1.034	0.537	1.000	0.421	1.000	0.353	1.000

MAE, mean absolute error; AE, absolute error; RMSE, root mean squared error.

^a^Independent variables: PF, RP, BP, GH, VT, SF, RE, MH.

^b^Independent variables: PF, BP, SF, RE, MH.

^c^Independent variables: PF, BP, GH, SF, RE, MH, PF squared, SF squared, RE squared.

^d^Independent variables: PCS, MCS, PCS × PCS, MCS × MCS, PCS × MCS.

### MNL performance

The MNL performances of the models are shown Table 4. Models 11, 12, 13, and 14 used the same independent variables as models 1, 2, 3, and 6, respectively. The pseudo R^2^ value of model 13 ranged between 0.455–0.615, which is slightly higher than models 11, 12 and 14. Proportions of estimation with absolute error > 0.05 or > 0.1 for MNL modeling of the derivation set were considerably lower that the OLS and two-part models, while the MAEs of the MNL models except model 12 were similar to the OLS and two-part models. Proportions of estimation > 0.05 in absolute error in MNL model in external validation decreased at around 45%, and it was lower than the OLS and two-part models.

**Table 4:** **Multinomial logistic modeling: predicting EQ-5D index scores from SF-36**

	**Model 11** ^**a**^		**Model 12** ^**b**^		**Model 13** ^**c**^		**Model 14** ^**d**^	
**Derivation set**								
R^2^	0.452–0.611		0.445–0.607		0.455–0.615		0.421–0.576	
MAE	0.088		0.121		0.084		0.099	
AE > 0.05 (%)	32.8		49.8		32.2		34.3	
AE > 0.1 (%)	25.0		45.5		23.7		26.0	
RMSE	0.297		0.347		0.290		0.315	
Predicted EQ-5D index								
Mean (SD)	0.826	(0.279)	0.731	0.310	0.826	(0.279)	0.829	(0.185)
Min/max	−0.171	/1.000	−0.171	1.000	−0.171	/1.000	−0.171	/1.000
**Internal validation set**								
MAE	0.097		0.119		0.092		0.101	
AE > 0.05(%)	35.9		48.5		37.2		35.9	
AE > 0.1(%)	28.7		43.2		28.9		28.1	
RMSE	0.312		0.344		0.304		0.315	
Predicted EQ-5D index								
Mean (SD)	0.825	(0.288)	0.732	0.313	0.821	(0.292)	0.828	(0.292)
Min/max	−0.171	/1.000	−0.171	1.000	−0.171	/1.000	−0.171	/1.000
**External validation set**								
MAE	0.085		0.125		0.075		0.084	
AE > 0.05 (%)	47.2		60.2		45.5		44.7	
AE > 0.1 (%)	26.0		52.0		26.0		26.8	
RMSE	0.292		0.354		0.273		0.290	
Predicted EQ-5D index								
Mean (SD)	0.883	(0.164)	0.753	0.204	0.892	(0.131)	0.877	(0.170)
Min/max	−0.171	/1.000	−0.171	1.000	0.151	/1.000	−0.171	/1.000

MAE, mean absolute error; AE, absolute error ; RMSE, root mean squared error.

^a^Independent variables: PF, RP, BP, GH, VT, SF, RE, MH.

^b^Independent variables: PF, BP, SF, RE, MH.

^c^Independent variables: PF, BP, GH, SF, RE, MH, PF squared, SF squared, RE squared.

^d^Independent variables: PCS, MCS, PCS × PCS, MCS × MCS, PCS × MCS.

**Figure 1:**
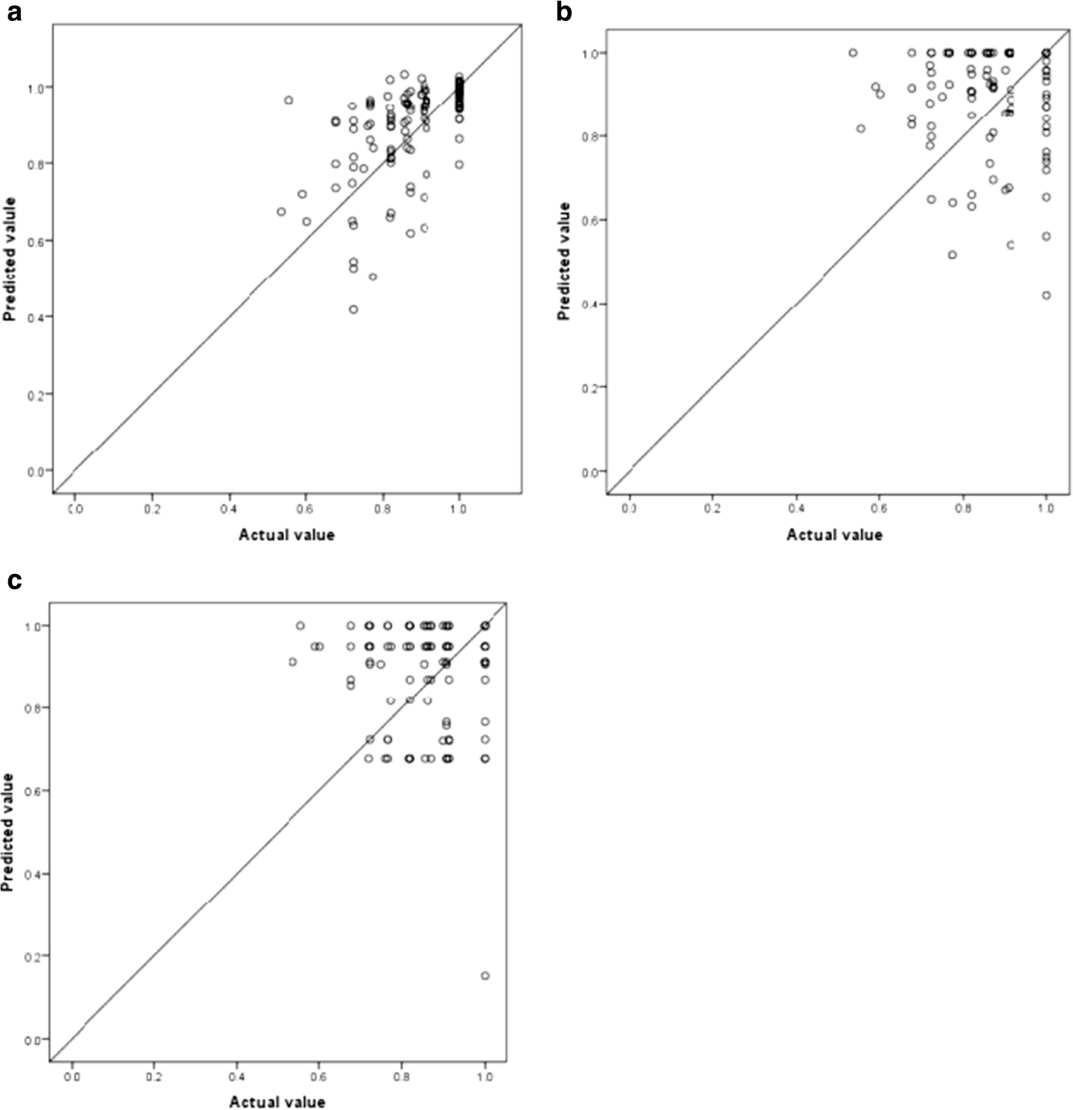
**Scatter plot of predicted values versus the actual EQ-5D index in the external validation sample; (a) ordinary least square regression (model 3) (b) two-part model (model 9) (c) multinominal logistic model (model 13).**

We displayed scatter plot of predicted values versus the actual EQ-5D index in external validation sample in OLS (Model 3), two-part model (Model 9) and MNL model (Model 13) using same explanatory variables (Figure 1) We also compared mean predicted value between cancer patients with and without active chemotherapy in 3 different models (Table 5). Mean predicted value in OLS and MNL were closer to actual mean value than two-part model.

**Table 5:** **Mean actual value and predicted value between external validation samples with and without current chemotherapy in 3 different models**

	**Actual value**	**Predicted value**		
		**OLS** ^**a**^	**Two part model** ^**a**^	**MNL** ^**a**^
Current chemotherapy (N = 95)	0.855	0.873	0.887	0.871
No current chemotherapy(N = 28)	0.915	0.951	0.965	0.949

^a^Independent variables: PF, BP, GH, SF, RE, MH, PF squared, SF squared, RE squared.

## Discussion

SF-36 is one of the most frequently used HRQOL instruments, and the EQ-5D is a unique instrument with national tariffs that were developed for use in Korea. In this study, eight domain scores or two SF-36 summary measures were mapped onto EQ-5D utility scores using diverse model specifications. Our findings show that the three different scoring algorithms demonstrate similar performances in terms of MAE and R^2^ values. Considering familiarity and predictability, the OLS model (including PF, BP, SF, RE, MH, GH, PF squared, SF squared and RE squared) could be recommended as the final algorithm in this study.

Our findings are comparable with previously reported evidence. The MAEs for our OLS models demonstrate consistent results for both the derivation (0.087–0.109) and external validation sets (0.082–0.097). The MAE in Ara & Brazier’s study, which were determined using a similar methodology, was approximately 0.13 [15]. The MAE of the OLS model was 0.0746 according to Sullivan et al., who mapped the two SF-12 summary measures, squared terms, and demographic variables onto the US-based EQ-5D index [19]. The OLS models in our study determined R^2^ between 64% (Model 5) and 75% (Model 3 & 4) for the EQ-5D index. The reported variance in Ara & Brazier’s study, which used similar independent variables as our study, varied between 56–59% [15]. The explanatory power of OLS regression when mapping the two SF-12 summary measures onto the UK-based EQ-5D index was 62.9%, and 65.6% when mapping all 12 items of SF-12 onto the UK-based EQ-5D index [14]. RP, VT, and GH domain scores were the non-significant when applying the domain scores in OLS model. This pattern is very similar to Ara & Brazier’s findings [15].

Two-part modeling demonstrated worse predictive power in aspects of RMSE than OLS regression and model fit in the modeling was insensitive to the choice of independent variables among those sets considered in comparison with OLS regression. The MAE values of the MNL models ranged between 0.084–0.099 for the derivation set and 0.075–0.101 for the validation set. These values are similar or slightly lower than the OLS regression values. Gray et al. reported an MAE value of 0.11 for the derivation set and 0.12 for the validation set when mapping all SF-12 questions to EQ-5D [30]. The range of the actual EQ-5D index was −0.171–1.0. The MNL model covered the entire possible EQ-5D range of the derivation set, however OLS regression covered 61–85% and the two-part model covered 69–93%.

A review of eight longitudinal studies reported a mean minimal important difference (MID) value of 0.074 (range = −0.011–0.140) for the EQ-5D index [31]. However, we cautiously used the mapping algorithm used in this study after considering that the MAE magnitude of this study was slightly higher than the conventional MID value of the EQ-5D index, and there was substantial proportion of estimation > 0.1 in terms of absolute error especially when applying the algorithms to datasets that are likely to have very low utility values.

Our current study has several strengths. First, we used patients with a range of HRQOL severity, from stroke patients to the general population; thus, our mapping algorithm could be applied to assess patients with various conditions. We ran OLS model in each different patient groups. Three (PF, BP, MH) out of five coefficients in OLS model showed equal statistical significant and sign in three different populations, although interaction of only PF and RE between groups showed statistically significant in the derivation set.

Second, our model was validated using both internal and external validation sets. Third, our data-collection methods were consistent, although data were obtained from several different studies because the same research team was involved in multiple studies. However, our present study also had several limitations. First, our external validation set tended to be healthier than the derivation set. There is some evidence that the MAE value of patients in poor health is higher than patients in good health when converting other HRQOL instruments to the EQ-5D index. Thus, further external validation of patients with severe conditions would be useful for verifying these findings. Second, this study examined only three mapping techniques. We also did not consider interaction between EQ-5D dimensions assuming independency in the MNL model, and further evaluation of the model is needed considering interaction between dimensions. There are other methodologies, such as CLAD and probabilistic mapping techniques using Bayesian networks [20], that could also be used.

Mapping between HRQOL measures onto EQ-5D utilities should be considered at best second-best method directly collected EQ-5D values [28]. Uncertainties in health utilities derived from mapping algorithm tend to be underestimated. Chan et al. recently reported correction method for the underestimation of variance of mapping algorithm–derived health utility [32].

## Conclusion

Predictability of OLS, MNL, and Two-part model are similar in mapping between SF-36 and EQ-5D health utility scores. OLS methods seems to be appropriate in aspects of model predictability and convenient application compared with two part model and MNL in our study yet the method may not always accurately predict the EQ-5D for poor health states. Currently, there is no Korean valuation set for SF-6D. Although there are some limitations to these algorithms, mapping from SF-36 scores and EQ-5D index could be used in economic evaluation as well as in clinical research until social tariff of SF-6D will be developed.

## Abbreviations

BP: Bodily pain
CLAD: Censored least absolute deviation
EQ-5D: EuroQol-5 dimension
GH: General Health perception
HRQOL: Health-related quality of life
MAE: Mean absolute error
MCS: Mental component summary
MH: Mental health
MID: Minimal important difference
MNL: Multinomial logistic
OLS: Ordinary least-square
PCS: Physical component summary
PF: Physical function
QALYs: Quality-adjusted life years
RE: Role-emotional
RMSE: Root mean squared error
RP: Role-physical
SF: Social functioning
VT: Vitality
